# Comparison of peritoneal dialysis with hemodialysis on survival of diabetic patients with end-stage kidney disease: a meta-analysis of cohort studies

**DOI:** 10.1080/0886022X.2019.1625788

**Published:** 2019-06-20

**Authors:** Jing Xue, Huihui Li, Quan Zhou, Shiwu Wen, Qiaoling Zhou, Wenhang Chen

**Affiliations:** a Institute of Hospital Administration, Xiangya Hospital, Central South University, Changsha, Hunan, China;; b Department of Scientific Research, Xiangya Hospital, Central South University, Changsha, Hunan, China;; c Department of Nephrology, Xiangya Hospital, Central South University, Changsha, Hunan, China;; d Department of Science and Education, The First People’s Hospital of Changde City, Changde, Hunan, China;; e Department of Epidemiology and Community Medicine, University of Ottawa, Ottawa, Ontario, Canada;; f Clinical Epidemiology Program, Ottawa Hospital Research Institute, Ottawa, Ontario, Canada

**Keywords:** Hemodialysis, peritoneal dialysis, chronic kidney disease, diabetes, survival, meta-analysis

## Abstract

**Aim:** Renal replacement therapy was primary treatment for end stage kidney (ESRD) patients. Numbers of studies comparing peritoneal dialysis (PD) and hemodialysis (HD) yielded inconsistent results. The aim of this study was to assess the mortality risk between diabetic PD patients and those in HD.

**Methods:** We included cohort studies comparing the risk of death among diabetic ESRD patients who receiving peritoneal dialysis or hemodialysis by searching Medline and Embase. Overall estimates were calculated using the random-effects model.

**Results:** Seventeen studies were included in the meta-analyses. Mortality comparison between PD and HD in the diabetic ESRD patients showed PD significantly increased mortality rate (hazard ratio (HR) 1.20; 95% confidence interval (CI) 1.10–1.30; *I*^2^ = 89.1%). The overall HR using an intention-to-treat analysis was 1.23 with 95% CI (1.13 to 1.34). Meta-regression demonstrated PD patients from Asian country were associated with increase in mortality risk (coefficient = 0.270, SE = 0.112, *p* = .033).

**Limitation:** The high heterogeneity in our meta-analyses undermined the robustness of the findings.

**Conclusion:** ESRD patients with diabetes may benefit more from HD than PD.

## Introduction

Diabetes has become the most common cause of end-stage renal disease (ESRD) [1]. Over the past few decades, patients requiring renal replacement therapy have been rapidly increasing worldwide, which imposed a tremendous burden on both family and society. Compared to normal population, ESRD patients have significant higher mortality rate [2]. Renal transplantation is the optimal therapy for ESRD and limited by the available organs. The most common treatments for ESRD are hemodialysis (HD) and peritoneal dialysis (PD). It is estimated that more than 2.2 million patients receive dialysis globally in 2020 [3]. However, the comparison of survival rates between PD and HD are still controversial.

One randomized controlled trial (RCT) has been published so far to compare outcomes in PD or HD patients [4]. The 3-year mortality rate was comparable between the two groups. However, the fact that only 38 patients finally recruited in the trial probably gave it insufficient statistic power to identify the survival difference for PD versus HD. The recruitment problem implied the difficulties in conducting such RCT in the future, as most patients prefer to make their own decision instead of being randomized to a modality.

Increased risk of cardiovascular events was observed in patients who had both ESRD and diabetes [5]. Numbers of studies indicated that ESRD patients with diabetes suffer higher death risk than ESRD patients without diabetes [6–8]. Vascular access may difficult to achieve because of diabetes-related atherosclerotic calcification in HD while continuous exposure to high glucose load might contribute to cardiac compromise and glucose imbalance in PD. It is vital to address which modality is better regarding the impact on mortality of ESRD patients especially in patients with diabetes and to synthesize existing knowledge to inform clinical practice and health policy. In the absence of extensive RCT data to compare survival outcomes associated with HD versus PD, observational studies of preexisting cohorts have had to suffice. Therefore, in the present work, we performed a comprehensive meta-analysis to compare the mortality of PD and HD in ESRD patients with diabetes.

## Materials and methods

### Data source and search strategy

Medline, Embase databases were searched for relevant articles through search strategies provided by a university librarian with retrieval deadline of April 2019. Keywords and corresponding medical subject headings were terms describing ‘mortality’ or ‘survival’ or ‘death’, ‘diabetes’, ‘dialysis modality’, ‘hemodialysis’, and ‘peritoneal dialysis’. The reference lists of all eligible articles and recent reviews on the subject were scanned to identify further potential studies. No language restriction was applied in the search. The object was carried out according to Preferred Reporting Items for Systematic Reviews and Meta-Analyses (PRISMA) guidelines and the Meta-analysis of Observational Studies in Epidemiology (MOOSE) [9,10]. The protocol for this meta-analysis was registered with PROSPERO (Website: https://www.crd.york.ac.uk/PROSPERO; Registration number: CRD42018085852).

### Study selection and data extraction

We included cohort studies comparing the risk of death among ESRD patients with diabetes who underwent peritoneal dialysis or hemodialysis. The outcome we focused was mortality. Diabetes mellitus was considered either as being the cause of ESRD or a comorbidity. The outcome of interest was all-cause mortality after the initiation of dialysis therapy.

Studies were excluded if they: (1) reported no hazard ratio (HR) on mortality; (2) were supplement, abstract, comments, editorials or letters; (3) reported estimates on mortality with the same or overlapping data; (4) included only home HD patients in the HD group; (5) included only automated peritoneal dialysis (APD) patients or patients using icodextrin in the PD group.

The suitable studies with the largest number of cases or latest publication were selected to avoid duplication. No follow-up duration restrictions were applied. Two authors independently selected all relevant studies based on inclusion and exclusion criteria (W.H. Chen and J. Xue). The articles with discrepancies were resolved by consensus or reviewed by a third author (Q.L. Zhou). Afterward, the following data was extracted independently using standardized data extraction forms: the first author’s name, year of publication, country, the source of data or name of cohort, number of patients in both PD and HD groups, follow up duration, stratifying factors, survival estimates (hazard ratio). Discrepancies were solved by discussion.

### Quality assessment

The Newcastle-Ottawa scale was used for quality appraisal of included studies by two authors (W.H. Chen and J. Xue) independently [11]. The scale had three main domains with quality score: the selection of the study groups (0–4 points), the comparability of the groups (0–2 points), and the ascertainment of outcome (0–3 points). A ‘high’ or ‘unclear’ risk of bias was scored ‘0’ while a ‘low’ risk of bias was scored ‘1’. A score above five points (including 5) was deemed as high quality.

### Statistical analysis

Meta-analyses were conducted using STATA (STATA version 13.0, StataCorp LP, College Station, TX, USA). All survival or mortality estimates for stratifications in individual studies were collected. A random-effects model was applied considering the confounding in observational studies. Overall HRs and 95% confidence intervals for mortality rates were calculated. For studies that only provided relevant data of subgroups (i.e., separate estimates for women and men), we combined within-study survival estimates using random-effects method for further meta-analysis. Between-study heterogeneity across studies was estimated by the *I*^2^ statistic [12]. The estimates corresponding to the longest follow up duration were selected for meta-analysis of comparison between HD and PD dialysis. We conducted meta-regression analyses and subgroup analyses to explore the heterogeneity. We performed sensitivity analyses by omitting one single study from the overall pooled analysis each time to evaluate the stability of the results. Publication bias was assessed by Egger’s test [13].

## Results

**Figure 1.**
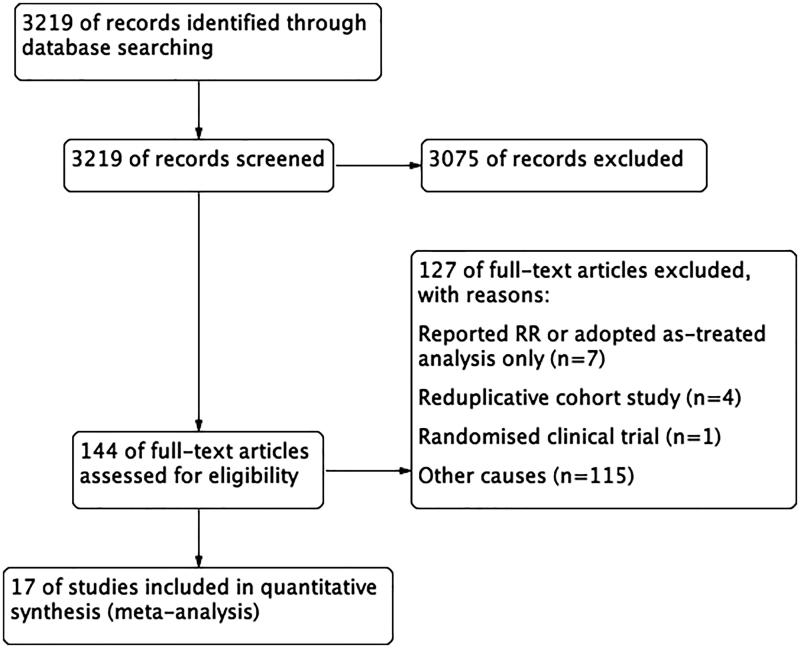
Study flow diagram.

The flow chart of the literature search and study selection was presented in Figure 1. A total of 3219 potential relevant studies without duplication were searched from both Medline and Embase databases. After title and abstract evaluation, 3075 irrelevant studies were excluded. The left 144 publications underwent further review while 127 of them were removed. Among the studies being removed, seven studies reported RR only [14–20], four were reduplicative cohort study [21–24], one was randomized clinical trial [4], the remain were excluded for other causes, such as no comparison groups, no relevant outcomes or no results of interest reported. Finally, a total of 17 studies were included in our analyses [25–41].

### Characteristics of included studies

The characteristics of the studies and basic information of the subjects were listed in Table 1. Among them, four studies were from North America [33,35,37,41], six from Europe [26,27,31,32,36,39], six from Asia [25,28–30,38,40], and one from Oceania [34]. The studies were published between 2007 and 2019 and included a total of 504 304 dialysis patients among whom 62 462 patients were treated with peritoneal dialysis, 441 842 patients underwent hemodialysis. The number of patients enrolled in studies ranged from 230 to 340 280. The follow up duration was 15 months to as long as ten years.

**Table 1.** Characteristics of included studies.

Author	Publication year	Location	Database	PD patients	HD patients	Enrollment period (year)	Follow-up duration (year)
Chang	2013	Korea	Gachon University Gil Hospital	118	112	2000–2009	10
Couchoud	2007	France	The French Renal Epidemiology and Information Network (REIN) registry	NR	NR	2002–2005	2
Heaf	2014	Danmark	The Danish Nephrology Registry (DNR)	916	1822	1990–2010	6
Huang	2008	China, Taiwan	Taiwan Renal Registry	761	16388	1995–2002	10
Kim	2017	Korea	The Korean Health Insurance Review and Asessment Service (HIRA) database	3996	12190	2005–2008	2
Lee	2009	China, Taiwan	Chang Gung Memorial Hospital, Keelung, Taiwan	79	437	1991–2005	15
Liem	2007	Netherlands	The Dutch End-Stage Renal Disease Registry (RENINE)	928	1615	1987–2002	1.3
Luijtgaarden	2016	Europe	The European Renal Association–European Dialysis and Transplant Association (ERA-EDTA)	6769	24594	1993–2007	5
Lukowsky	2013	USA	The United States Renal Data System (USRDS), the DaVita database	747	13863	2001–2006	1.7
Marshall	2014	New Zealand	The Australian and New Zealand Dialysis and Transplant Registry (ANZDATA) Registry	404	246	1997–2011	3
Mehrotra	2011	USA	The United States Renal Data System (USRDS)	30270	310010	1996–2004	5
Mircescu	2014	Romania	The Romanian Renal Registry	194	1246	2008–2011	3
Nesrallah	2016	USA	The United States Renal Data System (USRDS)	768	768	2004–2011	1.9
Sung Woo Lee	2019	Korea	The National Health Insurance Service database (NHIS)	10370	44809	2004–2015	5
Waldum-Grevbo	2015	Norway	The Norwegian Renal Registry	200	209	2005–2012	5
Wang	2016	China, Taiwan	The National Health Insurance Research Database (NHIRD) of Taiwan	327	328	2000–2010	2.6–2.8
Yeates	2012	Canada	The Canadian Organ Replacement Register (CORR)	5615	13205	1991–2007	5

NR: Non-reported.

### Study quality

The assessment of risk of bias in the included studies is summarized in Supporting Information table. The overall quality of included studies was high with quality score from 6 to 9. However, the included studies may have high risk of allocation bias or selection bias due to their observational design.

### Mortality comparison between HD and PD in diabetic ESRD patients

**Figure 2.**
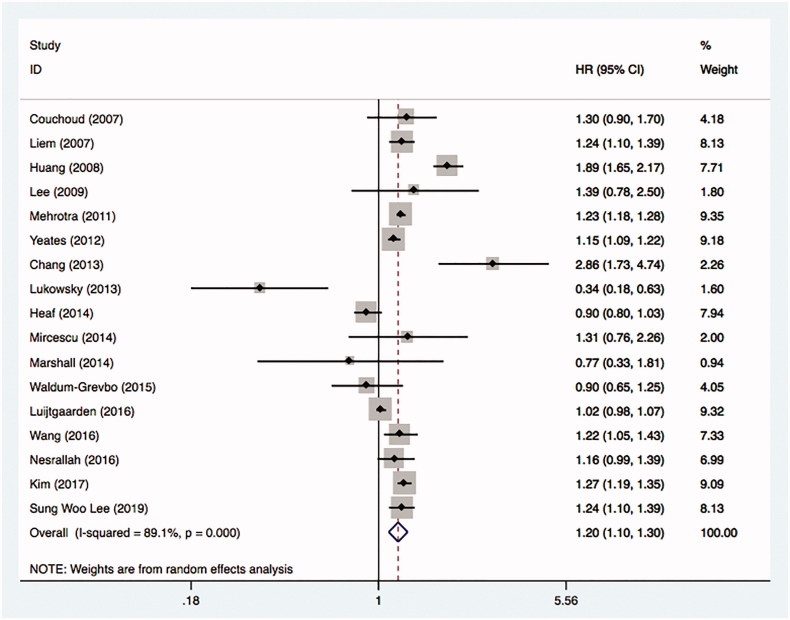
Comparative mortality of diabetic ESRD patients treated with HD and PD.

A total of 17 studies were included in the analysis. Figure 2 was the forest plot showed the combined results. The overall HR was 1.20 with 95% CI (1.10 to 1.30), which indicated hemodialysis had lower mortality risk than peritoneal dialysis in ESRD patients with diabetes. However, the heterogeneity between studies was high with the estimate for *I*^2^ equals to 89.1%.

**Figure 3.**
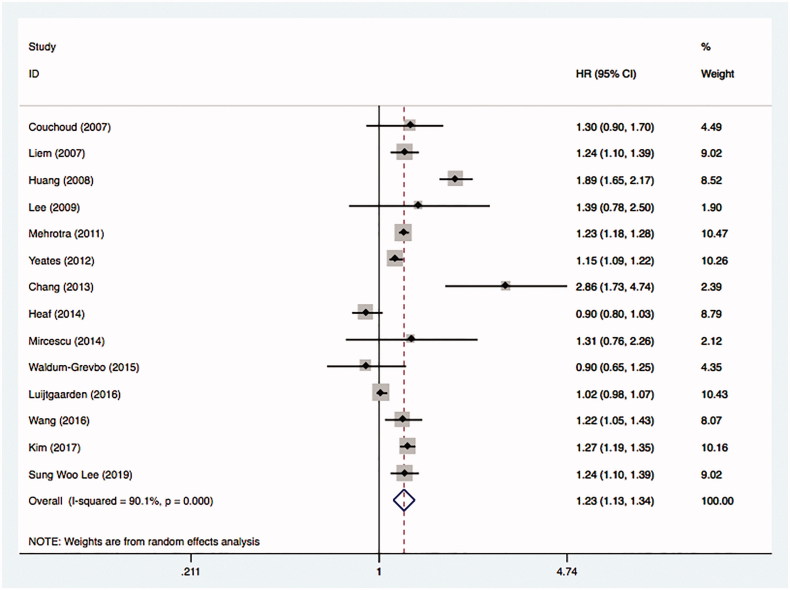
Comparative mortality of diabetic ESRD patients treated with HD and PD by intention-to-treat principle.

A total of 14 studies using an intention-to-treat framework were included in the analysis [25–32,35,36,38–41]. The comparison showed the overall HR was 1.23 with 95% CI (1.13 to 1.34). The heterogeneity between studies was significant with the estimate for *I*^2^ equals to 90.1% (Figure 3).

**Figure 4.**
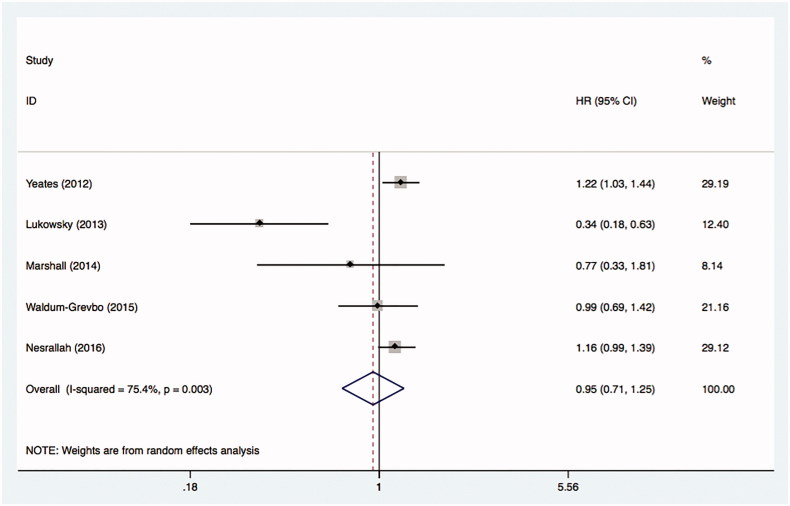
Comparative mortality of diabetic ESRD patients treated with HD and PD by as-treated principle.

A total of five studies using an as-treated framework were included in the analysis [33,34,37,39,41]. Figure 4 demonstrated the combined results was 0.95 with 95% CI (0.71 to 1.25). The estimate for *I*^2^ equals to 75.4%.

### Meta-regression and subgroup analyses

The high heterogeneity was presented in our meta-analysis. Meta-regression analyses were performed. Comparing with patients from non-Asian countries, PD patients from Asian countries were associated with increased mortality risk (coefficient = 0.270, SE = 0.112, *p* = .033). No significant association was observed in publication year (*p* = .245), duration of follow up period (*p* = .125).

**Figure 5.**
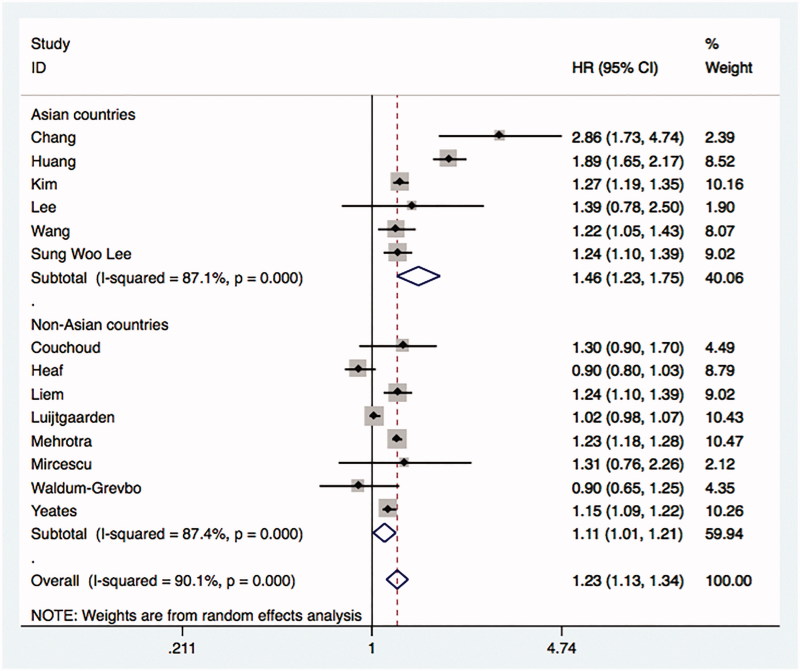
The summary estimates of subgroups by regions.

We did the subgroup analyses by regions to explore the potential sources of heterogeneity. The pooled HR of PD compared with HD was 1.46 (95% CI 1.23 to 1.75) in Asian countries while the HR was 1.11 (95% CI 1.01 to 1.21) in non-Asian countries. The heterogeneity was still significant in both two subgroups (Figure 5). The subgroup analyses by publication year and duration of follow up were seen in Supporting Information.

### Sensitivity analysis and publication bias

**Figure 6.**
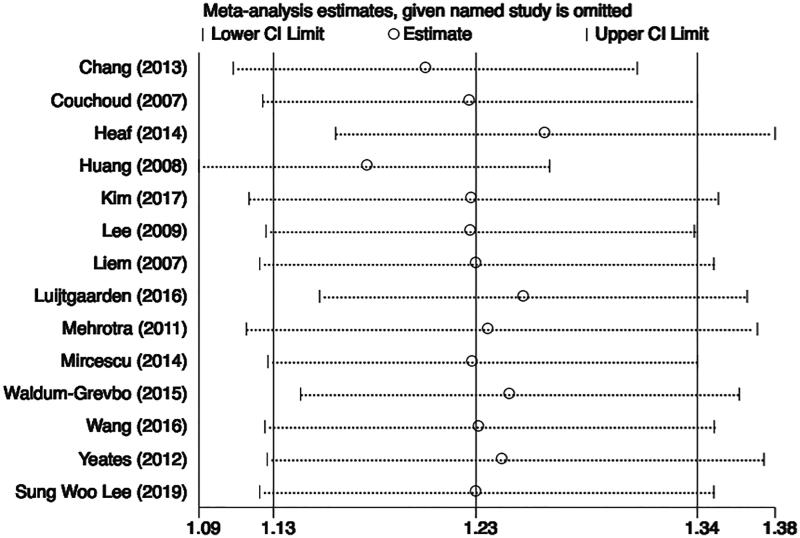
The sensitivity analysis.

**Figure 7.**
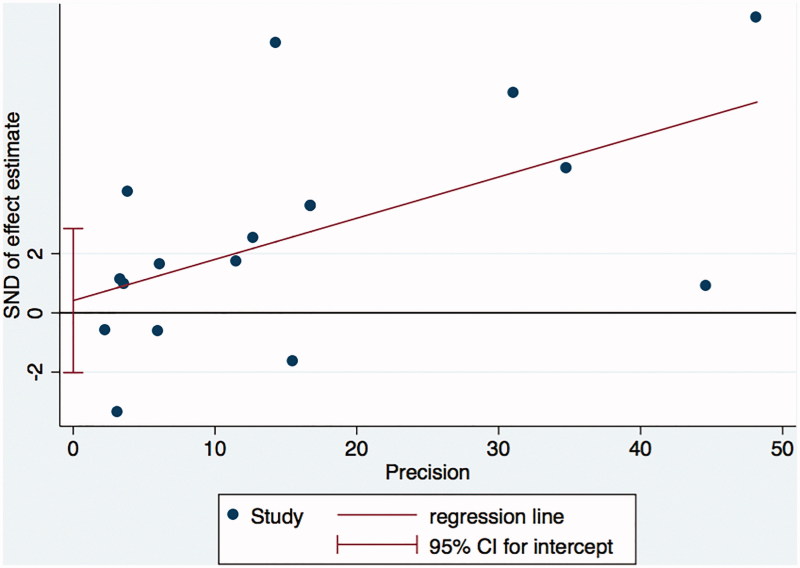
Publication bias using Egger test.

Sensitivity analysis showed the meta-analysis was low sensitivity and the overall results were stable and reliable (Figure 6). Egger test showed no publication bias (*p* = .722) (Figure 7).

## Discussion

The present systematic review and meta-analyses of 17 cohort studies found that HD dialysis might be superior to PD dialysis in diabetic ESRD patients. PD patients from Asian countries were probably associated with higher mortality risk comparing to PD patients from non-Asian countries.

NECOSAD study, which is the only RCT to date, failed to demonstrate a difference in mortality between PD and HD [4]. Difficulties in recruiting patients impede researchers to conduct a RCT with enough statistical power. Considering this, observational studies need to suffice. A previous meta-analysis by Han et al compared PD with HD in elderly ESRD patients [42]. The survival benefit from HD was significant in subgroup with diabetes. The pooled estimates comparing PD and HD for mortality was 1.26 (95% CI, 1.13–1.40) in elderly diabetic patients. The result was similar with ours. Furthermore, our studies indicate that HD showed lower mortality rate in ESRD patients with diabetes regardless of age. A review by Couchoud systematically discussed the available evidence concerning the modality comparison in diabetic patients with ESRD. The author argued it is not convincing to support a particular dialysis modality as first choice treatment in dialysis patients with diabetes [2]. Even though heterogeneity exists between studies, the results of meta-analyses could still provide us evidence to choose between HD and PD in diabetic patients.

In the subgroup analyses, we found diabetic PD patients from Asian countries had increased mortality risk comparing patients from non-Asian countries, which were mostly western countries. Studies showed no significant difference in survival between Asian and Caucasian PD patients [43,44]. A study by Cheng et al. compared mortality rate among HD patients in China and the US. The authors found Chinese HD patients had a survival benefit compared with patients in the US [45]. We assumed the higher HR of PD compared with HD in Asia could be the explained by the better survival benefit of HD in Asian. However due to the lack of related data in our analyses, we cannot explore this subject further.

When the data was pooled by analysis type (‘intention-to-treat’ or ‘as-treated’ analysis), we observed that HD shows a significant survival benefit over PD using intention-to-treat analysis while no difference in mortality was observed between the two groups by as-treated analysis. The reason for that maybe modality changes from PD to HD. A ‘PD first’ approach has been suggested for ESRD patients in many areas. Numbers of patients who initially went through PD turned to HD for various causes such as infections. These patients experienced increased mortality rate. Modality turnover and related increased death risk would lower the statistical power using as-treated analysis to detect the difference between the two groups. Death that ought to be in the PD group was assign to the HD group in as-treated analysis. Observing such phenomenon, we prefer the intention-to-treat analysis principle over as-treated analysis.

Those who need to start renal replacement therapy opt to consider the advantages, drawbacks and contradictions for each modality. PD needs no vascular access and provides continuous slow ultrafiltration. Some reports have suggested that residual renal function and urine output is better preserved among patients treated with PD as compared to HD [46,47], but it is still on debate. From economical view, PD costs approximately 30–40% less than HD and eases the burden on health care system [48]. As a result, ‘PD-First’ or ‘PD-Favored’ strategy was implemented for the ESRD patients in many areas [49–51]. However, PD might be associated with higher prevalence of infection, inadequate dialysis, inadequate volume control and catheter problems. All of the PD patients received mainly traditional glucose-based solutions, high in glucose degradation products (GDPs). GDPs were thought to induce apoptosis of peritoneal mesothelial cells [52]. Poor glycemic control is thought to associate with poor outcomes in dialysis patients [53,54]. Factors like inflammation, solution bioincompatibility, acidosis, or hyperglycemia were also affected by PD outcomes [55,56]. The diabetic population was reported to have higher risk of peritonitis, ultrafiltration failure, insulin resistance, worse glycemic control, and lower survival rate compared to non-diabetic patient on PD [57,58]. Above all, these mechanisms might explain our results, which suggested the higher mortality risk in diabetic PD patients compared with those in HD.

## Limitations

To the best of our knowledge, this is the first meta-analysis to assess the effect of dialysis modality on mortality in the diabetic ESRD patients. However, our study has some limitations. First, the included studies were observational design instead of randomized clinical trials. Patients were non-randomly assigned to either peritoneal dialysis or hemodialysis. Substantial confounders from selection and attrition bias may have impact on the outcomes. Even though most studies attempt to minimize the adverse effects through adjustment, unknown confounders or unmeasured confounders may still undermine the robustness of the outcomes. Second, the between study heterogeneity include patients’ profile, study year, statistical approach, follow up duration, and specific dialysis modality. There was great advance in PD therapy for diabetic patients after the availability of icodextrins, APD and biocompatible fluids [59,60]. Intensive hemodialysis or home hemodialysis had also been proposed with potential survival advantage over conventional hemodialysis [61]. However, most included studies compared all PD patients with HD patients irrespective of specific dialysis techniques. As a consequence, no subgroup analysis of specific dialysis techniques was performed. The majority of dialysis patients still using dextrose dialysate or underwent conventional hemodialysis, especially in developing countries. It is reasonable to believe that the conclusion we derived from the meta-analysis were mainly the comparisons between PD using dextrose dialysate with conventional hemodialysis. Third, medical and social context changed in the last decade. With the emergence of new dialysates, such as icodextrins or solutions using amino acid as osmotic agent, ultrafiltration capacity improved, the cardiovascular risk may be decreased in PD patients. Due to better patients’ education, related technology improved, new type of dialysis modality introduced (such as short daily hemodialysis), the mortality rate of either PD or HD may have changed over time. Considering factors above existed, it is explainable that some studies shows better survival benefit of HD while some studies may show opposite outcome.

In conclusion, HD might have a survival benefit in diabetic ESRD patients compared with PD. Without a large scale and well-organized randomized controlled trial exit, this meta-analysis provided comprehensive evidence for the patients or health care provider to choose between peritoneal dialysis and hemodialysis considering mortality. Further studies should focus on conducting prospective cohorts minimizing bias to the best extent possible.

## Supplementary Material

Supplementary Files
