# Supplementary material for: Comparison of peritoneal dialysis with hemodialysis on survival of diabetic patients with end-stage kidney disease: a meta-analysis of cohort studies
Source: Ren Fail. 2019 Jun 20;41(1):521–31. doi: 10.1080/0886022X.2019.1625788 (PMC6586097; doi:10.1080/0886022X.2019.1625788)
Supplement: Supplementary Files [file IRNF_A_1625788_SM0595.docx]

**Supplement table. Quality assessment of the included cohort studies**

| Study | Selection | | | | Comparability of cohorts | Outcome | | | Total score |
| --- | --- | --- | --- | --- | --- | --- | --- | --- | --- |
|  | Representativeness of exposed cohort | Selection of nonexposed cohort | Ascertainment of exposure | Outcome not present at baseline |  | Assessment of outcome | Sufficient follow-up duration | Adequate follow-up |  |
| Chang 2013 | 0 | 1 | 1 | 1 | 2 | 0 | 1 | 0 | 6 |
| Couchoud 2007 | 1 | 1 | 1 | 1 | 2 | 1 | 1 | 1 | 9 |
| Heaf 2014 | 1 | 1 | 1 | 1 | 2 | 1 | 1 | 1 | 9 |
| Huang 2008 | 1 | 1 | 1 | 0 | 1 | 1 | 1 | 1 | 7 |
| Kim 2017 | 1 | 1 | 1 | 1 | 2 | 1 | 1 | 1 | 9 |
| Lee 2009 | 0 | 1 | 1 | 1 | 1 | 1 | 1 | 0 | 6 |
| Liem 2007 | 1 | 1 | 1 | 1 | 1 | 1 | 1 | 1 | 8 |
| Luijtgaarden 2016 | 1 | 1 | 1 | 1 | 1 | 1 | 1 | 1 | 8 |
| Lukowsky 2013 | 1 | 1 | 1 | 1 | 2 | 1 | 1 | 1 | 9 |
| Marshall 2014 | 1 | 1 | 1 | 1 | 2 | 0 | 1 | 1 | 8 |
| Mehrotra 2011 | 1 | 1 | 1 | 1 | 2 | 1 | 1 | 1 | 9 |
| Mircescu 2014 | 1 | 1 | 1 | 1 | 1 | 1 | 1 | 1 | 8 |
| Nesrallah 2016 | 1 | 1 | 1 | 1 | 0 | 1 | 1 | 0 | 6 |
| Sung Woo Lee 2019 | 1 | 1 | 1 | 1 | 2 | 1 | 1 | 1 | 9 |
| Waldum-Grevbo 2015 | 1 | 1 | 1 | 1 | 2 | 1 | 1 | 0 | 8 |
| Wang 2016 | 1 | 1 | 1 | 1 | 2 | 1 | 1 | 1 | 9 |
| Yeates 2012 | 1 | 1 | 1 | 1 | 2 | 1 | 1 | 1 | 9 |

**
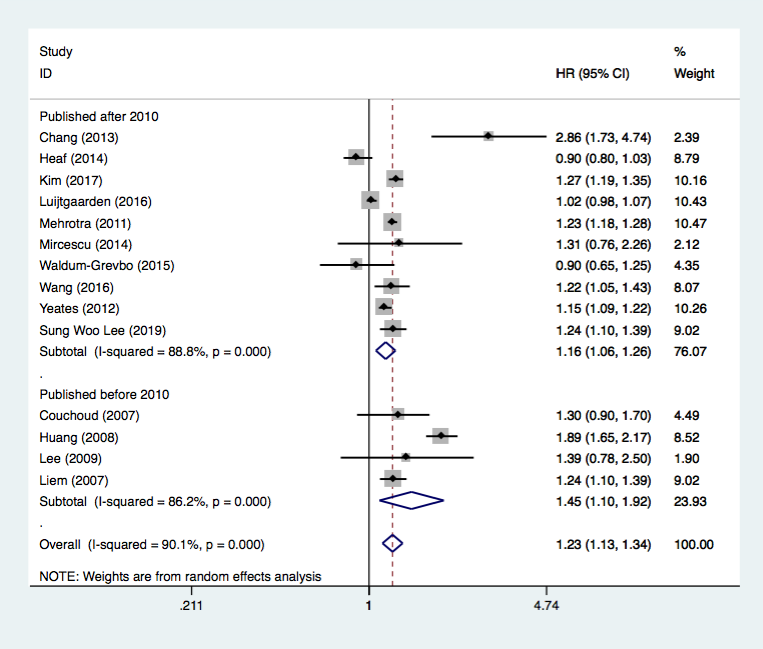
**

**Fig S1. The summary estimates of subgroups by publication years**

**
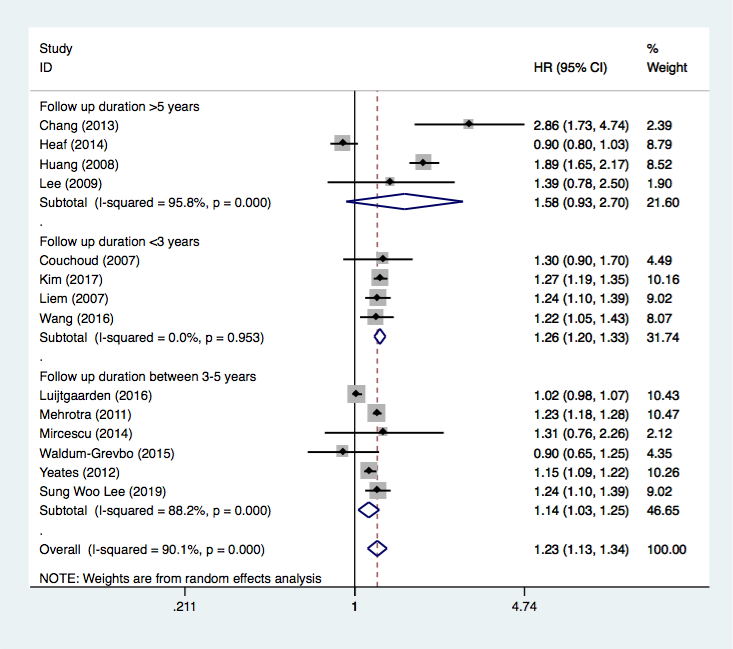
**

**Fig S2. The summary estimates of subgroups by follow up duration**

**Search strategy**

OVID Medline Embase

1. exp Renal Dialysis/

2. (hemodialysis or haemodialysis).tw.

3. (hemofiltration or haemofiltration).tw.

4. (hemodiafiltration or haemodiafiltration).tw.

5. exp Peritoneal Dialysis/

6. peritoneal dialysis.tw.

7. (CAPD or CCPD or APD).tw.

8. ((first or dialysis or choice or best) adj3 modality).tw.

9. ((first or dialysis or modality or starting or best) adj3 choice).tw.

10. ((dialysis or modality or best) adj3 start).tw.

11. ((begin or first or initiat$) adj3 dialysis).tw.

12. mortality/

13. mortality.mp.

14. survival.mp.

15. survival/

16. Survival Analysis/

17. Kidney Diseases/

18. exp Renal Replacement Therapy/

19. Renal Insufficiency/

20. exp Renal Insufficiency, Chronic/

21. (end-stage renal or end-stage kidney or endstage renal or endstage kidney).tw.

22. (ESRF or ESKF or ESRD or ESKD).tw.

23. (chronic kidney or chronic renal).tw.

24. or/17-23

25. or/12-16

26. or/8-11

27. or/5-7

28. or/1-4

29. (27 and 28) or 26

30. 24 and 25 and 29

31. exp diabetes mellitus/

32. Diabetic Nephropathies/

33. diabet$.tw.

34. or/31-33

35. 30 and 34
